# Trends of Multidrug-Resistant Gram-Negative Bacteria in Tamale Metropolis, Ghana (2020–2023)

**DOI:** 10.3390/antibiotics15050434

**Published:** 2026-04-27

**Authors:** Valentine Cheba Koyiri, Sang Sook Beck, Moonsoo Yoon, Abass Abdul Karim, Enoch Weikem Weyori, Bernard Nkrumah, Samuel Yaw Opoku, Joon Sup Yeom

**Affiliations:** 1 Graduate School of Public Health, Yonsei University, Seoul 03722, Republic of Korea; vkoyiri@yonsei.ac.kr; 2 Zonal Public Health Reference Laboratory, Tamale P.O. Box 99, Ghana; abass.komei@yahoo.com (A.A.K.); enoch.weikem-weyori@cug.edu.gh (E.W.W.); 3 Asian Institute for Bioethics and Health Law, General Complex B/D Yonsei University Health System, Seoul 03722, Republic of Korea; beck@yuhs.ac; 4 Institute of Tropical Medicine, College of Medicine, Yonsei University, Seoul 03722, Republic of Korea; msyoon1962@naver.com; 5 African Field Epidemiology Network, Accra, Ghana; bnkrumah@afenet.net; 6 Catholic University of Ghana Fiapre, Sunyani P.O. Box 363, Ghana; syopoku58@gmail.com; 7 Division of Infectious Disease, Department of Internal Medicine, Severance Hospital, Yonsei University College of Medicine, 50-1 Yonsei-ro Seodaemun-gu, Seoul 03722, Republic of Korea

**Keywords:** multidrug resistance, Gram-negative bacteria, antimicrobial resistance, Tamale metropolis, public health, infection control

## Abstract

**Background/Objectives**: Multidrug-resistant (MDR) Gram-negative bacteria represent a significant public health concern worldwide, particularly in resource-limited settings. In Ghana’s Tamale Metropolis, limited data exist on the prevalence and trends of MDR bacteria, posing challenges to effective antimicrobial stewardship. **Methods**: This study analyzed microbiological data from 2020 to 2023 to address these knowledge gaps. **Results**: Among the 4859 clinical samples analyzed, 1570 (33.7%) yielded Gram-negative bacterial isolates, with an MDR prevalence of 40.6%. The most frequently isolated organisms were *Klebsiella* spp. (28.9%) and *Escherichia coli* (20.4%). Resistance to cephalosporins (51%) and ciprofloxacin (46%) was particularly pronounced, highlighting the diminishing efficacy of commonly used antibiotics. Older adults (aged 60 years and above) presented the highest MDR prevalence, reflecting the vulnerability of this demographic group. **Conclusion**:These findings underscore the urgent need for enhanced antimicrobial stewardship programs, improved infection prevention and control measures, and continuous resistance monitoring to combat the growing threat of MDR bacteria in the region. Strengthening laboratory capacity and adherence to strict antibiotic usage policies are crucial for reducing the burden of MDR infections and improving patient outcomes.

## 1. Introduction

The rapid rise in multidrug-resistant (MDR) Gram-negative bacteria represents a major global public health threat, undermining the effectiveness of current antimicrobial therapies and contributing to increased morbidity, mortality, and healthcare costs [1,2]. The World Health Organization has warned of a potential “post-antibiotic era,” in which common infections and minor injuries could become life-threatening due to ineffective treatment [3]. This crisis is particularly severe in low- and middle-income countries (LMICs), where factors such as widespread antibiotic misuse, self-medication, poor-quality antimicrobials, and weak regulatory enforcement accelerate the emergence and spread of antimicrobial resistance [4].

In Ghana, as in many LMICs, significant challenges persist in addressing antimicrobial resistance (AMR), including limited surveillance systems, inadequate laboratory capacity, and enforcement of antimicrobial use policies [5]. In the northern region of Ghana, particularly the Tamale Metropolis, high rates of antibiotic misuse and resistance have been reported, especially at referral facilities such as the Tamale Teaching Hospital, where incomplete treatment courses and inappropriate prescribing practices are common [6,7].

Despite these concerns, there remains a lack of data on the prevalence, resistance patterns, and temporal trends of MDR Gram-negative bacteria in this region. The absence of robust, context-specific evidence limits the ability to design and implement effective antimicrobial stewardship and public health interventions.

This study addresses these gaps by assessing the prevalence and trends of MDR Gram-negative bacteria in the Tamale Metropolis from 2020 to 2023. The findings are expected to inform policymakers, strengthen laboratory and surveillance systems, and support targeted infection prevention and antimicrobial stewardship strategies in resource-limited settings.

## 2. Result

### 2.1. Demographic Characteristics

Table 1 presents the demographic distribution of clinical cases of suspected bacterial infection at the Zonal Public Health Reference Laboratory between 2020 and 2023. A total of 4859 clinically suspected cases were recorded during the study period, of which 1570 (32.3%) were confirmed as Gram-negative bacterial infections. In addition, fungal infections (n = 413, 8.5%) and Gram-positive organisms (n = 172, 3.5%) were identified, while the majority of samples showed no bacterial growth. Among the Gram-negative isolates, 933 (59.4%) were not multidrug-resistant. Multidrug-resistant (MDR) cases were more prevalent among individuals aged ≥60 years (22.9%) and females (n = 361, 56.7%). The highest proportion of Gram-negative isolates was recorded in 2020 (n = 489, 31.1%). MDR was more frequently observed among inpatient cases (n = 356, 55.9%) compared to outpatients. Statistically significant associations were observed between MDR status and sex, patient status, and year of isolation. However, no significant association was found between age category and MDR status.

### 2.2. Laboratory Markers

The distribution of multidrug-resistant (MDR) and non-MDR Gram-negative organisms varied across specimen types, as shown in Table 2. Among Gram-negative isolates from blood samples, 5.3% were MDR, while 3.6% were non-MDR. Gastrointestinal samples exhibited the highest proportion of MDR isolates (80.0%). Respiratory specimens accounted for the largest proportion of Gram-negative isolates (61.8%), followed by urine (17.5%), wound swabs (8.3%), and high vaginal swabs (HVS) (6.1%).

*Klebsiella* spp. (30.19%) were the most frequently isolated Gram-negative organisms, whereas *Enterobacter* spp. (4.97%) were the least common. Other significant isolates included *Escherichia coli* (20.4%), *Moraxella catarrhalis* (21.34%), and *Pseudomonas* spp. (13.63%).

Antimicrobial resistance patterns showed high resistance to cephalosporins (51.0%), ciprofloxacin (46.0%), aminoglycosides (34.0%), and amoxicillin–clavulanate (31.7%). In contrast, lower resistance rates were observed for chloramphenicol (0.6%), tetracycline (4.3%), and meropenem (12.9%).

Statistical analysis revealed significant associations between specimen type and the prevalence of Gram-negative organisms, as well as between Gram-negative status and resistance to specific antimicrobials, such as aminoglycosides.

### 2.3. Period Prevalence of MDR Among Gram-Negative Confirmed Patients

**Figure 1:**
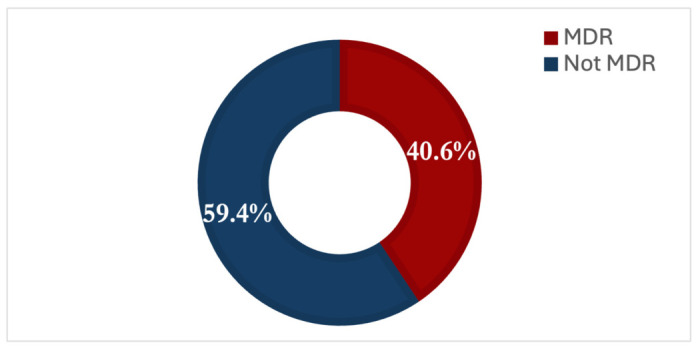
Multidrug resistance prevalence in isolated organisms.

The estimated period prevalence of multidrug resistance (MDR) among Gram-negative bacterial isolates from 2020 to 2023 was 40.6%, as shown in Figure 1. This high prevalence highlights the increasing difficulty in managing Gram-negative infections due to resistance to multiple classes of antimicrobial agents. The findings underscore the need for strengthened antimicrobial stewardship and targeted interventions to address the declining effectiveness of available treatment options against MDR pathogens.

### 2.4. Trends in MDR Patients Across Demographic Characteristics

Trend analysis indicated an overall increase in multidrug resistance (MDR) among Gram-negative bacterial isolates from 2020 to 2023. The prevalence rose from 168 (34.4%) in 2020 to 167 (45.4%) in 2023. A marked spike occurred in 2021 of 163 (43.3%), followed by a slight decline in 2022 of 139 (41.4%), before an increasing trend in 2023.

**Figure 2:**
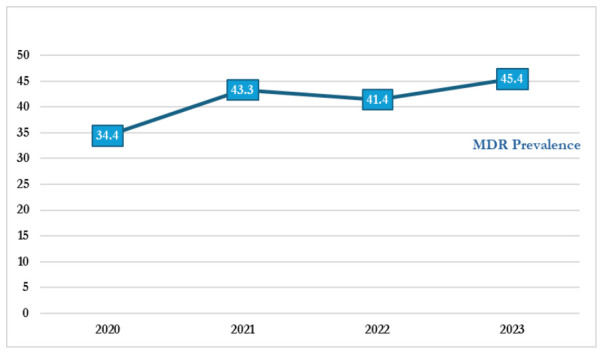
Trend of multidrug resistance across the study years.

The overall increase in MDR prevalence over the study period highlights the growing burden of antimicrobial resistance in the region. The slight decline observed in 2022 may reflect temporal variation in resistance patterns or shifts in underlying epidemiological dynamics. These findings underscore the need for sustained surveillance and targeted antimicrobial stewardship interventions to curb the rising trend of MDR. The temporal trend is illustrated in Figure 2.

### 2.5. Trends in MDR Prevalence of Isolated Gram-Negative Bacterial Species

Trends in MDR prevalence among Gram-negative bacterial species from 2020 to 2023 revealed distinct patterns. Enterobacteriaceae remained the predominant group across all years, peaking in 2021 and showing a renewed increase in 2023. *Acinetobacter* spp. demonstrated an overall increase in prevalence up to 2021, followed by a decline in subsequent years. *Pseudomonas* spp. exhibited the most marked increase in MDR prevalence between 2020 and 2022, indicating an emerging concern, although a slight decline was observed in 2023.

**Figure 3:**
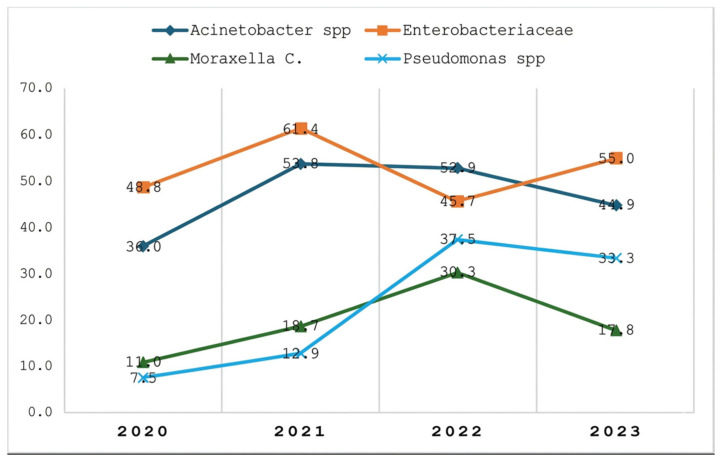
Trend of MDR cases in isolated Gram-negative organisms across the years.

Similarly, the prevalence of *Moraxella catarrhalis* peaked in 2022 before declining in 2023. Overall, Enterobacteriaceae and *Acinetobacter* spp. maintained relatively high MDR prevalence, whereas *Pseudomonas* spp. showed notable year-to-year variability. These trends highlight the dynamic nature of antimicrobial resistance and underscore the need for continuous surveillance and targeted interventions. The species-specific trends are presented in Figure 3.

### 2.6. Trends in MDR Patients by Gender

From 2020 to 2023, trends in multidrug-resistant (MDR) Gram-negative bacterial infections showed variation by sex. In 2020, females accounted for a higher proportion of MDR cases (63.7%) compared to males (36.3%). In 2021, this pattern reversed, with males accounting for 52.2% of cases and females 47.9%.

**Figure 4:**
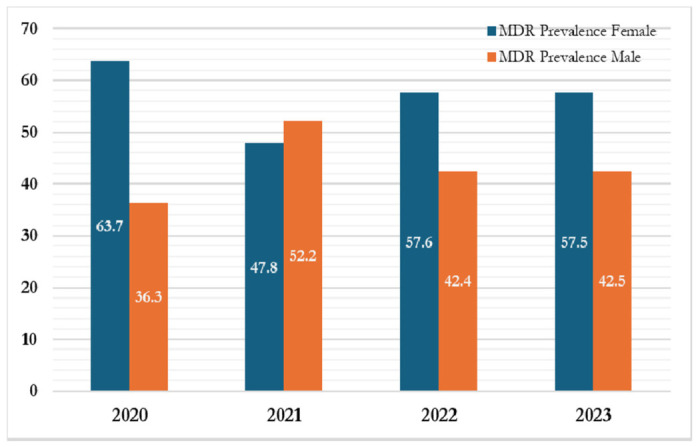
Annual Patterns of MDR Distribution by Sex.

In 2022, the proportion of MDR cases among females increased to 57.6%, while that among males decreased to 42.4%. A similar pattern was observed in 2023, with females accounting for 57.5% of cases and males 42.5%. These findings indicate temporal variation in the sex distribution of MDR cases and highlight the need for further investigation into potential sex-related factors influencing antimicrobial resistance patterns. The distribution by sex is illustrated in Figure 4.

### 2.7. Sex Distribution of MDR Cases Across Sample Types

The distribution of multidrug-resistant (MDR) Gram-negative infections varied by sex across different sample types. Females accounted for the majority of MDR cases in blood 22 (66.7%), gastrointestinal tract (GIT) and high vaginal swab (HVS) samples 4 (100%), urine 105 (69.5%), and wound samples 36 (54.6%).

In contrast, there was a higher proportion of MDR cases in respiratory samples from males, 184 (55.4%), than in those categorized as “others”, 5 (71.4%). While female patients consistently dominated most sample types, respiratory and “other” samples highlighted a notable predominance of male cases.

**Figure 5:**
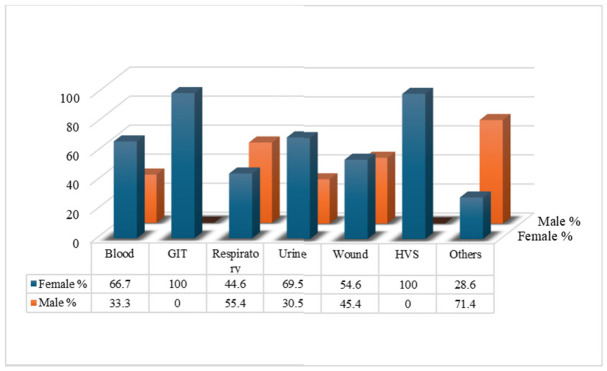
Distribution of Gram-Negative MDR Cases by Sex and Sample Type.

These findings underscore the need to explore sex-specific factors influencing MDR prevalence across different clinical specimens Figure 5.

### 2.8. Sex Distribution of MDR Patients by Organism Type

The distribution of multidrug-resistant (MDR) Gram-negative infections by organism type showed variation by sex. Males accounted for a slightly higher proportion of MDR infections caused by *Pseudomonas* spp. 24 (52.2%) and *Moraxella catarrhalis* 26 (52.9%). In contrast, Enterobacteriaceae infections were more common among females, who accounted for 278 (59.5%) of MDR cases compared to 189 (40.5%) among males.

**Figure 6:**
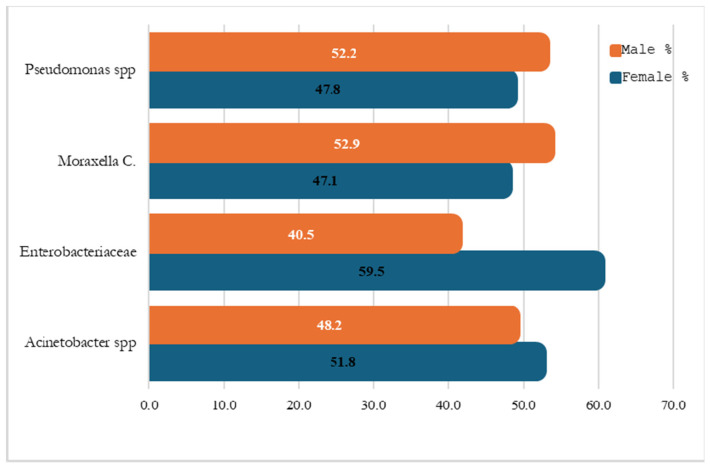
Distribution of Gram-Negative MDR Cases by Sex and Organism Type.

For *Acinetobacter* spp., the distribution was relatively balanced, with 24 (48.2%) of cases occurring in males and 29 (51.8%) in females. The greatest sex-related difference was observed among Enterobacteriaceae infections, where females represented a higher proportion of MDR cases, as seen in Figure 6.

### 2.9. Age Distribution of MDR Patients

The distribution of multidrug-resistant (MDR) cases across age groups from 2020 to 2023 revealed notable variations. The overall MDR prevalence was estimated at 40.8%. Individuals aged 60 years and above consistently accounted for the highest proportion of MDR cases, except in 2023, when the 46–60 age group had the highest prevalence, 35 (21.0%).

The proportion of MDR cases among individuals aged <5 years increased from 13 (7.7%) in 2020 to 24 (14.4%) in 2023. Similarly, the 46–60 age group showed an increasing trend, rising from 28 (16.7%) to 35 (21.0%) over the study period. In contrast, the 5–14 age group consistently had the lowest proportion of MDR cases across all years.

Although MDR prevalence showed a slight decline between 2020 and 2022, it increased again in 2023. Overall, older age groups appear to be disproportionately affected by MDR infections, underscoring the need for targeted interventions in high-risk populations. The age-specific distribution is presented in Table 3.

### 2.10. Trends of Antimicrobial Resistance Patterns for Antimicrobial Categories

From 2020 to 2023, antimicrobial resistance trends varied across six major antibiotic classes: cephalosporins (n = 836), aminoglycosides (n = 557), azithromycin (n = 380), meropenem (n = 211), sulfonamides (n = 318), and amoxicillin–clavulanate (n = 520). Detailed annual resistance patterns across these antimicrobial classes are presented in Table 4. Resistance to cephalosporins increased markedly from 39.1% in 2020 to 58.6% in 2022, followed by a slight decline in 2023. Aminoglycoside resistance decreased to 30.9% in both 2021 and 2022 but rose sharply to 56.2% in 2023. Resistance to azithromycin showed some fluctuation, with a notable decline to 12.6% in 2023.

Meropenem resistance demonstrated a steady increase, rising from 4.7% in 2020 to 20.0% in 2023. Sulfonamide resistance peaked at 31.8% in 2022 before decreasing to 21.8% in 2023. Resistance to amoxicillin–clavulanate declined in 2021 and 2022 but increased again to 20.0% in 2023.

Overall, these trends indicate increasing resistance across several key antibiotic classes, particularly cephalosporins, aminoglycosides, and carbapenems, highlighting the need for strengthened antimicrobial stewardship and continuous resistance surveillance.

### 2.11. Antimicrobial Resistance Patterns Across Gram-Negative Organisms

Antimicrobial resistance (AMR) patterns varied across Gram-negative bacterial species, as summarized in Table 5. *Acinetobacter* spp. showed the highest resistance to ciprofloxacin (49.6%) and the lowest resistance to amikacin (10.7%). Similarly, *Enterobacter* spp. exhibited high resistance to amoxicillin–clavulanate (61.5%) and ciprofloxacin (51.3%), while resistance to meropenem (3.8%) and amikacin (10.3%) was comparatively low.

*Escherichia coli* demonstrated high resistance to amoxicillin–clavulanate (46.6%) and ciprofloxacin (45.7%), with lower resistance to meropenem (8.1%) and amikacin (11.0%). Likewise, *Klebsiella* spp. showed substantial resistance to amoxicillin–clavulanate (49.2%) and ciprofloxacin (42.2%), whereas resistance to meropenem (9.1%) and amikacin (8.0%) remained low.

*Moraxella catarrhalis* exhibited the highest resistance to ciprofloxacin (56.0%) and ceftazidime (31.6%), and the lowest resistance to amoxicillin–clavulanate (1.4%) and meropenem (17.0%). *Pseudomonas* spp. showed high resistance to ciprofloxacin (35.7%) and gentamicin (30.5%), with lower resistance to amikacin (9.9%) and meropenem (24.4%).

Overall, amikacin and meropenem consistently demonstrated lower resistance rates across most Gram-negative organisms, suggesting retained effectiveness. In contrast, ciprofloxacin and amoxicillin–clavulanate showed relatively high resistance rates, particularly among *Klebsiella* spp., *Enterobacter* spp., and *Escherichia coli*, indicating reduced efficacy of these agents.

### 2.12. Proportion of MDR Bacteria Among Isolated Gram-Negative Bacteria

The prevalence of multidrug resistance (MDR) among isolated Gram-negative bacteria from 2020 to 2023 varied across species. *Enterobacter* spp. exhibited the highest MDR proportion (57.7%), defined as resistance to three or more classes of antimicrobial agents. High MDR rates were also observed in *Escherichia coli* (52.2%) and *Klebsiella* spp. (52.1%).

*Acinetobacter* spp. demonstrated a moderate MDR prevalence of 46.3%. In contrast, other Gram-negative organisms, including *Moraxella catarrhalis* and *Pseudomonas* spp., showed lower MDR rates of 19.5% and 21.5%, respectively.

**Figure 7:**
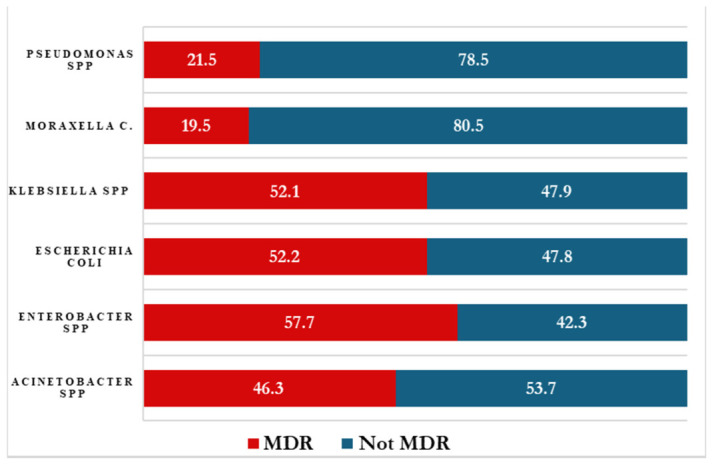
Antimicrobial resistance for multidrug resistance across isolated Gram-negative bacteria.

These findings indicate a substantial resistance burden among key pathogens such as *Enterobacter* spp., *Escherichia coli*, and *Klebsiella* spp., underscoring the need for targeted antimicrobial stewardship interventions to limit the spread of MDR organisms. The species-specific distribution of MDR is presented in Figure 7.

### 2.13. Poisson Regression Analysis of Temporal Trends in MDR Cases by Age Group and Sex

A multivariable Poisson regression model with robust standard errors was fitted to assess temporal trends in multidrug-resistant (MDR) cases from 2020 to 2023, adjusting for age group and sex. The analysis demonstrated a statistically significant decline in MDR cases over time, with each additional year associated with a 12% reduction in MDR incidence (IRR = 0.88; 95% CI: 0.82–0.95; *p* < 0.001). This corresponds to an average annual percentage change (AAPC) of −12%, indicating substantial progress in MDR control over the study period, particularly between 2020 and 2022, although descriptive trends suggest a plateau in 2023.

Age-specific effects revealed significant heterogeneity in MDR burden. Compared with children under five years, individuals aged 60 years and above had more than twice the risk of MDR (IRR = 2.15; 95% CI: 1.51–3.05; *p* < 0.001), representing the highest burden. Similarly, individuals aged 26–35 years (IRR = 1.92; *p* < 0.001) and 46–60 years (IRR = 1.88; *p* = 0.001) exhibited significantly elevated risks, while those aged 15–25 years showed a moderate increase in risk (IRR = 1.48; *p* = 0.039). In contrast, children aged 5–14 years had significantly lower risk (IRR = 0.52; *p* = 0.011). These findings indicate a clear age gradient, with MDR burden increasing with age and peaking among older adults and economically productive populations.

Sex-disaggregated analysis further showed that females had a significantly higher MDR burden compared to males, with a 31% increased risk (IRR = 1.31; 95% CI: 1.12–1.54; *p* = 0.002). Importantly, interaction analyses revealed significant effect modification by both age and sex. The year-by-age interaction (IRR = 1.23; *p* < 0.05) indicates that the temporal decline in MDR was not uniform across age groups, with some groups experiencing faster reductions and others exhibiting persistent or fluctuating trends. Similarly, the year-by-sex interaction (IRR = 1.11; *p* = 0.023) suggests that temporal changes differed between males and females, with evidence pointing toward a slower decline or relative persistence of MDR burden among females.

Overall, these findings highlight a significant downward temporal trend in MDR cases, accompanied by substantial age- and sex-related disparities. While the declining trend suggests improvements in MDR control efforts, the observed heterogeneity and plateauing pattern in later years underscore the need for targeted, population-specific interventions to sustain progress and prevent resurgence as illustrated in Table 6.

## 3. Discussion

Epidemiological and laboratory surveillance are essential for the effective management of bacterial infections and the control of antimicrobial resistance (AMR), particularly in low- and middle-income countries [8]. The substantial burden of multidrug-resistant (MDR) Gram-negative infections observed in this study highlights critical gaps in diagnostic capacity, infection prevention, and antimicrobial stewardship in Ghana.

The observed MDR prevalence of 40.6% indicates a significant and persistent threat to clinical management and health system performance, consistent with findings from previous studies in Ghana [9,10]. For public health policy, this underscores the urgent need to strengthen national AMR surveillance systems through improved laboratory infrastructure, standardized data reporting, and integration of facility-level data into national surveillance platforms [8]. Enhancing the capacity of regional and zonal laboratories is essential for generating timely and reliable resistance data to guide evidence-based decision-making.

**Figure 8:**
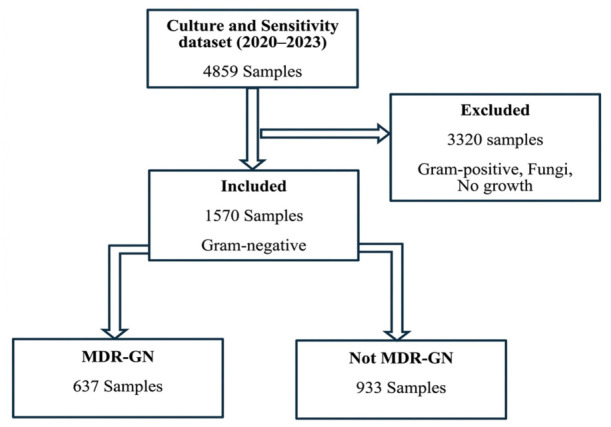
Sampling Procedure.

The high resistance observed to cephalosporins, ciprofloxacin, and aminoglycosides (Table 4 and Table 5) is of particular concern because these agents are included in the World Health Organization classification of Critically Important Antimicrobials for Human Medicine [11]. The increasing resistance to these agents observed in this study, particularly among *Escherichia coli*, *Klebsiella* spp., and *Enterobacter* spp. (Table 5; Figure 8), therefore has important clinical and public health implications, as it may substantially limit the availability of effective treatment options.

The higher MDR burden observed among females and older adults is consistent with known epidemiological patterns, including the high incidence of urinary tract infections among women and increased healthcare exposure among the elderly [7]. These findings highlight the need for targeted interventions within high-risk populations. Strengthening infection prevention and control (IPC) measures in healthcare facilities, particularly for hospitalized patients, is critical [8]. In addition, antimicrobial stewardship programs should be expanded beyond tertiary facilities to include primary and secondary healthcare levels, where inappropriate prescribing and over-the-counter antibiotic use are more prevalent [8]. The relatively low MDR observed in *Pseudomonas* spp. may reflect lower antibiotic pressure, differences in sample composition, or local prescribing practices [12]. It may also be influenced by the types of infections captured or laboratory testing approaches. Continuous surveillance is essential to monitor potential changes in resistance patterns [13].

At the community level, the findings emphasize the importance of enforcing regulations on antibiotic sales and reducing self-medication practices, which are key drivers of AMR in LMICs [4,8]. Public education campaigns aimed at improving awareness of appropriate antibiotic use should be prioritized and aligned with Ghana’s National Action Plan on AMR [8].

The Poisson regression indicates a modest but significant annual decline in MDR incidence, suggesting some progress in control efforts. However, the plateau observed in 2023 implies that these improvements may not be sustained and require strengthening. A clear age gradient was observed, with older adults and individuals aged 26–60 years showing higher MDR risk. This likely reflects increased healthcare exposure and antibiotic use, emphasizing the need for age-specific interventions [14]. Females exhibited a significantly higher MDR risk, possibly due to greater healthcare utilization and infection patterns such as UTIs [15]. The differential temporal trends by sex highlight the need for gender-sensitive AMR strategies. The significant interaction between time and age indicates that MDR trends vary across age groups, with some populations experiencing slower declines [16]. This underscores the importance of targeted, population-specific interventions rather than uniform approaches.

Overall, the rising prevalence of MDR Gram-negative bacteria necessitates a coordinated policy response that integrates surveillance, antimicrobial stewardship, infection prevention, and public education. Strengthening these components is critical to mitigating the impact of AMR on morbidity, mortality, and healthcare costs in Ghana.

## 4. Methods

### 4.1. Study Design

This study employed a retrospective cross-sectional design to analyze microbiological data from the Zonal Public Health Reference Laboratory (ZPHRL) covering the period from 1 January 2020 to 31 December 2023. De-identified laboratory records were accessed for research purposes between 1 December 2025 and 5 December 2025. Data were obtained from the WHO.net database and included results of bacterial isolation and antimicrobial susceptibility testing. The retrospective design enabled the examination of trends in Gram-negative bacterial infections, antimicrobial resistance patterns, and associated clinical characteristics over the study period.

### 4.2. Study Population

The study population comprised patients from healthcare facilities in the Tamale Metropolis who underwent microbiological testing at the Zonal Public Health Reference Laboratory (ZPHRL) between 1 January 2020 and 31 December 2023. The dataset included individuals across diverse demographic groups, including different age categories, sexes, and socioeconomic backgrounds. Both inpatient and outpatient specimens were analyzed to provide a comprehensive assessment of multidrug-resistant Gram-negative bacterial infections in the urban setting.

### 4.3. Study Setting

The study utilized data from the Zonal Public Health Reference Laboratory (ZPHRL), a regional public health reference laboratory serving northern Ghana. The facility is ISO 9001-certified and operates a Biosafety Level II molecular laboratory equipped with advanced tools for molecular diagnostics and antimicrobial resistance research. ZPHRL serves as a key center for public health investigations and is currently pursuing ISO 15189 accreditation.

### 4.4. Sampling Procedure

#### 4.4.1. Sampling Frame

The sampling frame consisted of bacteriology records documented in the ZPHRL register and stored electronically in the WHO.net database. This database served as the basis for selecting Gram-negative bacterial isolates that met the study’s objectives. The overall sampling process is illustrated in Figure 8, which outlines the selection of Gram-negative bacterial isolates from 1 January 2020 to 31 January 2023, based on predefined inclusion and exclusion criteria.

#### 4.4.2. Sampling Technique

Purposive sampling was used to select Gram-negative bacterial isolates from 1 January 2020 to 31 December 2023. This non-probability sampling technique enabled the selection of cases that met predefined inclusion criteria, ensuring that only isolates relevant to the study objectives were included.

#### 4.4.3. Inclusion Criteria

The study included clinical samples (blood, sputum, urine, and wound swabs) that were processed for bacterial culture and antimicrobial susceptibility testing, from which Gram-negative bacteria were isolated. The target organisms included *Escherichia coli*, *Klebsiella* spp., *Enterobacter* spp., *Pseudomonas* spp., *Acinetobacter* spp., and *Moraxella catarrhalis*.

#### 4.4.4. Exclusion Criteria

Samples were excluded if they had incomplete records, showed no bacterial growth, or yielded Gram-positive bacteria or fungal isolates.

#### 4.4.5. Sample Size Estimation

No predetermined or computed sample size was used for this study. Among the 4859 clinical samples processed between 1 January 2020 and 31 December 2023, 1570 were identified as Gram-negative bacterial infections after applying the exclusion criteria. A post hoc power analysis was performed via G*Power statistical software v 3.1.9.7, which estimated a statistical power of 95% for the observed effect size.

### 4.5. Antibiotic Susceptibility Testing, Identification, and Isolation of Bacteria

Routine microbiological analysis of clinical samples was conducted at the Zonal Public Health Reference Laboratory (ZPHRL) in accordance with standard operating procedures. Gram-negative bacterial isolates were identified based on Gram staining characteristics, conventional biochemical tests (including catalase, oxidase, urease, and substrate utilization assays), and, where applicable, the API 20E identification system (bioMérieux SA, Marcy-l’Étoile, France).

Antimicrobial susceptibility testing was performed using the disk diffusion method. Inhibition zone diameters were measured and interpreted in accordance with Clinical and Laboratory Standards Institute (CLSI) guidelines and reported in millimeters. The antibiotics tested included amikacin (AMK), azithromycin (AZM), cefotaxime (CTX), ceftazidime (CAZ), ceftriaxone (CRO), chloramphenicol (CHL), ciprofloxacin (CIP), gentamicin (GEN), meropenem (MEM), penicillin V (PEN), tetracycline (TET), and trimethoprim–sulfamethoxazole (SXT).

Following culture, isolation, identification, and antimicrobial susceptibility testing, results were recorded in the laboratory’s electronic microbiology database. Data entry was verified by the chief data manager and a laboratory technician to ensure accuracy.

### 4.6. Variables

The primary outcome variable was multidrug resistance (MDR), classified as either MDR or non-MDR, defined as resistance to three or more classes of antimicrobial agents. Independent variables included age, sex, patient status (inpatient or outpatient), specimen type (e.g., blood, stool, urine), and the specific Gram-negative bacterial isolate (e.g., *Escherichia coli*, *Klebsiella* spp., and others).

#### Multidrug Resistance

Multidrug resistance (MDR) was defined as a binary categorical variable, classified as MDR or non-MDR, in accordance with the standardized definitions proposed by the European Centre for Disease Prevention and Control (ECDC) and the Centers for Disease Control and Prevention (CDC). MDR was operationally defined as non-susceptibility to at least one agent in three or more antimicrobial classes, while non-MDR referred to isolates that did not meet this criterion.

### 4.7. Statistical Analysis

Data analysis was performed using Stata version 17 (StataCorp LLC, College Station, TX, USA). Descriptive statistics were used to summarize the data, and results were presented using tables and figures. The chi-square test was applied to assess associations between categorical variables. Only complete cases were included in the analysis; records with missing data were excluded to ensure data integrity and analytical validity.

### 4.8. Ethical Consideration

Permission to access the data was obtained from the Ghana Health Service. The study adhered to established ethical standards, ensuring data confidentiality and compliance with applicable guidelines. Ethical approval was granted by the Committee on Human Research, Publications and Ethics of Kwame Nkrumah University of Science and Technology (KNUST) (Ref: CHRPE/AP/204/26; approved on 3 February 2026).

## 5. Conclusions

This study highlights the alarming prevalence (40.6%) and increasing trends of multidrug-resistant (MDR) Gram-negative bacteria in the Tamale Metropolis from 2020 to 2023. Increasing resistance to key antibiotics, including cephalosporins, aminoglycosides, and carbapenems, threatens effective treatment options, particularly for infections caused by *Klebsiella* spp. and *Escherichia coli*. Vulnerable populations, including older adults, females, and children under five years of age, are disproportionately affected. These findings emphasize the urgent need for improved antimicrobial stewardship, enhanced laboratory surveillance, and infection prevention measures to address the growing antimicrobial resistance (AMR) crisis.

## Figures and Tables

**Table 1:** Demographic characteristics of the study participants.

Variables	Group of Isolated Organisms		Total N = 1570	X^2^ (*p* Value)
	MDR N = 637	Not MDR N = 933		
Age grouping of patients				(0.062)
<5	45 (7.1)	40 (4.3)	85 (5.4)	
5–14	23 (3.6)	38 (4.1)	61 (3.9)	
15–25	91 (14.3)	125 (13.4)	216 (13.7)	
26–35	123 (19.3)	221 (23.7)	344 (21.9)	
36–45	88 (13.8)	140 (15.0)	228 (14.5)	
46–60	121 (19.0)	188 (20.2)	309 (19.7)	
60+	146 (22.9)	181 (19.4)	327 (20.8)	
Gender of patient				(0.004)
Female	361 (56.7)	460 (49.3)	821 (52.3)	
Male	276 (43.3)	473 (50.7)	749 (47.7)	
Patient Status				(<0.001)
Outpatient	281 (44.1)	559 (59.9)	840 (53.5)	
Inpatient	356 (55.9)	374 (40.1)	730 (46.5)	
Year of specimen				(0.006)
2020	168 (34.35)	321 (65.64)	489 (31.15)	
2021	163 (43.24)	214 (56.76)	377 (24.01)	
2022	139 (41.37)	197 (58.63)	336 (21.40)	
2023	167 (45.38)	201 (54.62)	368 (23.44)	

**Table 2:** Laboratory markers of the study participants.

Variables	Group of Isolated Organisms		Total N = 1570	X^2^ (*p* Value)
	MDR N = 637	Not MDR N = 933		
Specimen type collected				(<0.01)
Blood	33 (49.25)	34 (50.7)	67 (4.27)	
GIT	4 (80.0)	1 (20.0)	5 (0.32)	
Respiratory	332 (34.23)	638 (65.77)	970 (61.78)	
Urine	151 (54.91)	124 (45.09)	275 (17.52)	
Wound	66 (50.38)	65 (49.62)	131 (8.34)	
High Vaginal Swab	44 (46.32)	51 (53.68)	95 (6.05)	
Others	7 (25.93)	20 (74.07)	27 (1.72)	
Organisms isolated				(<0.01)
*Acinetobacter* spp.	56 (46.28)	65 (53.72)	121 (7.71)	
*Enterobacter* spp.	45 (57.69)	33 (42.31)	78 (4.97)	
*Escherichia coli*	175 (52.24)	160 (47.76)	335 (21.34)	
*Klebsiella* spp.	247 (52.11)	227 (47.89)	474 (30.19)	
*Moraxella catarrhalis*	68 (10.7)	280 (30.0)	348 (22.17)	
*Pseudomonas* spp.	46 (19.54)	168 (80.46)	214 (13.63)	
Antimicrobial category				(0.035)
Aminoglycoside	658 (41.9)	912 (58.1)	1570	
Amoxicillin-Clavulanate	766 (48.8)	804 (51.2)	1570	
Meropenem	546 (34.8)	1024 (65.2)	1570	
Cephalosporins	725 (46.2)	845 (53.8)	1570	
Ciprofloxacin	763 (48.6)	807 (51.4)	1570	
Azithromycin	552 (35.2)	1018 (64.8)	1570	
Sulfamethoxazole	699 (44.5)	871 (55.5)	1570	
Chloramphenicol	639 (40.7)	931 (59.3)	1570	
Tetracycline	646 (41.1)	924 (58.9)	1570	

**Table 3:** Trends of MDR cases according to age-specific categorization.

Age Grouping	Trend Years of MDR				Total [N = 637]
	2020 [n = 168]	2021 [n = 163]	2022 [n = 139]	2023 [n = 167]	
Under 5	13 (7.7)	2 (1.2)	6 (4.3)	24 (14.4)	45 (7.1)
5–14	7 (4.2)	5 (3.1)	2 (1.4)	9 (5.4)	23 (3.6)
15–25	19 (11.3)	23 (14.1)	23 (16.6)	26 (15.6)	91 (14.3)
26–35	27 (16.1)	36 (22.1)	30 (21.6)	30 (18.0)	123 (19.3)
36–45	34 (20.2)	21 (12.9)	17 (12.2)	16 (9.6)	88 (13.8)
46–60	28 (16.7)	33 (20.3)	25 (18.0)	35 (21.0)	121 (19.0)
60+	40 (23.8)	43 (26.4)	36 (25.9)	27 (16.2)	146 (22.9)
MDR prevalence	34.5	23.1	20.7	21.7	40.8

**Table 4:** Antibiotic profile for antimicrobial resistance across the study years.

Year of Case	Antimicrobial Category					
	Cephalosporin [n = 836]	Aminoglycoside [n = 557]	Azithromycin [n = 380]	Meropenem [n = 211]	Sulfoamide [n = 318]	Amoxicilllin-Clavulanate [n = 520]
2020	201 (39.1)	159 (30.9)	128 (24.9)	24 (4.7)	28 (5.5)	240 (46.7)
2021	215 (53.6)	100 (24.9)	112 (27.9)	56 (14.0)	98 (24.4)	179 (44.6)
2022	201 (58.6)	84 (24.5)	92 (26.8)	55 (16.0)	109 (31.8)	24 (7.0)
2023	219 (57.5)	214 (56.2)	48 (12.6)	76 (20.0)	83 (21.8)	77 (20.2)
Resistance (%)	51.0	34.0	23.2	12.9	19.4	31.7

**Table 5:** AMR patterns of Gram-negative organisms isolated over the study period.

Antibiotics	*Acinetobacter* spp. [n = 121]	*Enterobacter* spp. [n = 79]	*E. coli* [n = 335]	*Klebsiella* spp. [n = 474]	*Moraxella c.* [n = 348]	*Pseudomanas* spp. [n = 213]
Amikacin	13 (10.7)	8 (10.3)	37 (11.0)	38 (8.0)	104 (29.9)	21 (9.9)
Amoxiclav	35 (28.9)	48 (61.5)	156 (46.6)	233 (49.2)	5 (1.4)	8 (3.8)
Azithromycin	21 (17.4)	21 (26.9)	116 (34.6)	152 (32.1)	32 (9.2)	15 (7.0)
Ceftazidime	56 (46.3)	31 (39.7)	127 (37.9)	187 (39.5)	110 (31.6)	46 (21.6)
Ciprofloxacin	60 (49.6)	40 (51.3	153 (45.7)	200 (42.2)	195 (56.0)	76 (35.7)
Ceftriaxone	45 (37.2)	22 (28.2)	121 (36.1)	153 (32.3)	7 (2.0)	19 (8.9)
Cefotaxime	30 (24.8)	23 (29.5)	108 (32.2)	144 (30.4)	26 (7.5)	9 (4.2)
Gentamicin	45 (37.2)	15 (19.2)	95 (28.4)	118 (24.9)	81 (23.3)	65 (30.5)
Meropenem	22 (18.2)	3 (3.8)	27 (8.1)	43 (9.1)	59 (17.0)	52 (24.4)
Sulfamethoxazole	32 (26.4)	18 (23.1)	88 (26.3)	126 (26.6)	11 (6.3)	11 (5.2)

**Table 6:** Poisson Regression Analysis of Temporal Trends in MDR Cases by Age Group and Sex.

Variable	Category	IRR (Incidence Rate Ratio)	Robust SE	95% CI	*p*-Value
Year (trend)	Per year increase	0.88	0.033	0.82–0.95	<0.001
Age Group	Under 5	–	–	–	–
	5–14	0.52	0.128	0.31–0.86	0.011
	15–25	1.48	0.278	1.02–2.15	0.039
	26–35	1.92	0.365	1.34–2.76	<0.001
	36–45	1.37	0.261	0.94–1.99	0.102
	46–60	1.88	0.352	1.30–2.71	0.001
	60+	2.15	0.401	1.51–3.05	<0.001
Sex	Male	–	–	–	–
	Female	1.31	0.105	1.12–1.54	0.002
Year × Age	interaction	1.23	0.032	1.09–2.01	<0.05
Year × Sex	interaction	1.11	0.102	1.07–3.31	0.023

## Data Availability

The original contributions presented in this study are included in the article. Further inquiries can be directed to the corresponding author.

## Abbreviations

MDR	Multidrug resistance
AMR	Antimicrobial Resistance
ZPHRL	Zonal Public Health Reference Laboratory
LMICs	Low- and middle-income countries
ISO	International Organization for Standardization
CLSI	Clinical and Laboratory Standards Institute
UTIs	Urinary tract infections
ESBL	Extended-spectrum beta-lactamase
*E. coli*	*Escherichia coli*
GIT	Gastrointestinal tract
HVS	High Vaginal Swab
